# Exploring Subtypes Based on Depression and Anxiety in Preoperative Patients With Carpal Tunnel Syndrome: A Two-Step Cluster Analysis

**DOI:** 10.7759/cureus.80928

**Published:** 2025-03-20

**Authors:** Akihito Yoshida, Katsuyuki Iwatsuki, Takaaki Shinohara, Hitoshi Hirata

**Affiliations:** 1 Department of Integrated Health Sciences, Graduate School of Medicine, Nagoya University, Nagoya City, JPN; 2 Department of Hand Surgery, Graduate School of Medicine, Nagoya University, Nagoya City, JPN; 3 Department of Orthopaedic Surgery, Daido Byoin, Nagoya City, JPN

**Keywords:** anxiety, carpal tunnel syndrome, cluster analysis, depression, preoperative period

## Abstract

Background

Although a growing body of literature supports the importance of depression and anxiety, the assessment of these modifiable factors has not been considered in recent clinical practice guidelines for patients with carpal tunnel syndrome (CTS). This study aimed to classify patients with CTS into preoperative subgroups using cluster analysis based on the Japanese versions of the Self-Rating Depression Scale (SDS) and Pain Anxiety Symptom Scale-20 (PASS-20). Outcome measures were also compared for each cluster.

Methods

Data from 65 patients with CTS were analyzed. The SDS and PASS-20 psychological parameters were grouped using the K-means cluster method, according to Ward’s method. Sociodemographic, disease-related, physical, psychological, and disability outcomes were compared between the clusters.

Results

A three-cluster solution, which categorized patients into “psychologically normal,” “only depression,” and “psychologically impaired” clusters, was selected. Upper extremity disability in the “psychologically impaired” cluster was more severe compared to that in other clusters.

Conclusions

We provided evidence for the detection of depression and anxiety in patients with CTS at a preoperative period.

## Introduction

Carpal tunnel syndrome (CTS) is the most common entrapment neuropathy [1]. It is caused by compression of the median nerve at the carpal tunnel, an anatomical region delimited superiorly by the transverse carpal ligament and inferiorly by the carpal bones [2]. Edema, tendon inflammation, hormonal changes, and manual activity associated with the median nerve and digital flexor tendons in the carpal tunnel contribute to nerve compression and dysfunction [3]. Literature shows that both conservative and surgical treatments have clinical benefits for CTS [4]. A guideline published by a multidisciplinary group of experts suggests that the first step in the treatment of CTS is conservative. Surgical treatment should be reserved for patients with advanced compression [5].

A recent meta-analysis identified depression and anxiety as important predictors of poor postoperative outcomes after carpal tunnel release (CTR), but these predictors do not always appear [6]. Therefore, these factors should be evaluated by physicians, physiotherapists, and occupational therapists [7]. Although a growing body of literature supports the importance of depression and anxiety as modifiable factors in CTR [8], the assessment of these factors has not been considered in recent clinical practice guidelines for patients with CTS [9]. As most patients may end up requiring surgery [10], a better understanding of psychological factors before CTR could also help in choosing more appropriate treatments.

The relationship between CTS and depression is particularly interesting. Both conditions are highly prevalent, particularly in women [11]. Moreover, some patients with CTS awaiting surgery may present with depression, which is related to a low level of physical activity [12]. Symptoms of depression were associated with higher severity of symptoms in 71% of the studies that considered this prognostic factor, followed by symptoms of anxiety (66%) [6]. We hypothesized that the population would be divided into three groups based on the presence of depression and anxiety with pain: 1) normal, 2) single impairment (only depression), and 3) double impairment. In musculoskeletal diseases, identified mental health factors (symptoms of anxiety and depression) have been reported to be relevant to optimizing post-surgical outcomes. Previous systematic reviews have shown that these factors are associated with poorer postoperative outcomes in shoulder surgery, spine surgery, knee replacement, and CTR [6]. However, the subgrouping of mental health factors and the relevance of upper extremity disability before operation remain under discussion. This retrospective cross-sectional study aimed to classify patients with CTS into preoperative subgroups using cluster analysis according to the presence of depression and anxiety. Outcome measures were also compared for each cluster. Additionally, we aimed to determine the clinical features of each cluster in terms of demographics, physical functions, and disabilities. This study was conducted according to the Strengthening the Reporting of Observational Studies in Epidemiology (STROBE) statement.

## Materials and methods

Patients and study design

We collected the data for 67 patients with idiopathic CTS who underwent CTR in this cross-sectional study. This study was designed as a sub-analysis of a prospective study. All patients were treated at a university hospital between December 2012 and November 2018. Adult patients aged ≥18 years before surgery were recruited for this study. Two patients were excluded because of Parkinson disease and syringomyelia following Arnold-Chiari malformations. No patients had a medical history of depression and anxiety. Thus, the clinical data of 65 patients were analyzed. Patients were diagnosed with CTS by a hand surgeon based on clinical and electrophysiological findings. All patients met the following criteria: a history of paresthesia in the distribution of the median nerve; a positive result in a provocative test such as the Phalen wrist flexion test, carpal tunnel compression test, and Tinel-like sign; and a positive result on electrophysiological examination [13]. This study was approved by the Ethical Review Committee of a University Hospital (approval number: 2021-0351) and complied with all provisions of the Declaration of Helsinki. The informed consent was obtained in writing from all participants.

Variables assessed

We assessed all variables two weeks before the operation. Twenty parameters, including subscale scores, were collected. The parameters included sociodemographic, disease-related, physical, psychological, and disability outcomes.

The sociodemographic outcomes included age and sex. Disease-related outcomes included symptom duration, operative side, affected side, and Padua classification. The Padua classification reflects the neurophysiological severity of median nerve impairment based on the results of nerve-conduction studies [14]. Physical variables included grip strength using the Jamar Hydraulic Hand Dynamometer (Lafayette Instrument Company, Lafayette, IN) [15], key pinch strength using the Hydraulic Pinch Gauge (Baseline Inc., Boise, ID), pulp-pinch strength, tactile threshold using the Semmes-Weinstein monofilament test using the Semmes-Weinstein monofilaments (Patterson Medical, Saint Paul, MN) [16], static two-point discrimination sensation using the Disk-Criminator (AliMed, Dedham, MA) [17], visual analog scale (VAS) score for pain, and numerical rating scale (NRS) score for numbness. The VAS is a simple assessment tool consisting of a 10-cm unmarked line with zero, representing no pain, on one end, and 10, representing the worst pain ever experienced, on the other, with patients indicating the severity of their pain to the researcher. NRS can provide a measure of the severity of numbness on the basis of the patient’s verbal responses to an 11-step evaluation in which 0 and 10 reflect no and the worst possible numbness, respectively. Physical outcome data obtained at the side scheduled for surgery were used for all analyses.

The psychological outcomes included the score for the Japanese version of the Self-Rating Depression Scale (SDS) [18] and the Japanese version of the Pain Anxiety Symptom Scale-20 (PASS-20) [19]. SDS is designed to assess the level of depression [18]. It is a short self-administered survey to quantify the depressed status of a patient. Therefore, it has been widely used in populations of various ages to screen for depression with validity and reliability, including the Japanese version. The SDS consists of 20 items that evaluate the affective, psychological, and somatic symptoms associated with depression. These include 10 positively worded and 10 negatively worded questions. Each question is scored on a Likert scale ranging from one to four (with the ratings corresponding to the replies “1 = rarely,” “2 = some of the time,” “3 = a good part of the time,” and “4 = most of the time”). The overall SDS score ranged from 20 to 80 and could be categorized into four ranges: normal range, less than 40 points; mildly depressed, 40-47 points; moderately depressed, 48-55 points; and severely depressed, 56 points and above. In this study, the cutoff point of 40 points was used in terms of screening for depression in the Japanese [18]. PASS-20 is a 20-item self-reported measure to assess pain-related anxiety [18]. The scale contains four subscales: cognitive anxiety, escape/avoidance, fear, and physiological anxiety. Each item is rated on a six-point scale ranging from zero (never) to five (always). The total score ranged from zero to 100, with higher scores representing greater anxiety regarding pain. In this study, the scores calculated to be above the mean values for Japanese patients were considered to indicate the presence of anxiety (i.e., the mean scores were total score, 37.8; cognitive anxiety, 11.3; escape/avoidance, 10.8; fear, 8.5; and physiological anxiety, 7.2). The questionnaire has good reliability, validity, and internal consistency [19].

The disability outcome was the Hand10 score, which was evaluated using 10 short, easy-to-understand questions and explanatory illustrations [20]. The total score ranged from zero to 100, with higher scores representing greater upper extremity disabilities. Hand10 scores have shown high acceptability and reliability even among elderly individuals because of the use of explanatory illustrations.

Statistical analysis

Descriptive statistics were initially used to summarize the collected data regarding sociodemographic, disease-related, physical, psychological, and disability outcomes. Among sociodemographic and disease-related outcomes, age and symptom duration were analyzed using ratio scales. The Padua classification was analyzed as an ordinal scale. Sex, operative side, and affected side were analyzed using nominal scales. Among the physical, psychological, and disability outcomes, grip strength, key pinch strength, pulp-pinch strength, static two-point discrimination sensation, VAS score for pain, NRS score for numbness, and SDS, PASS-20, and Hand10 scores were analyzed as ratio scales. The tactile threshold was analyzed as an ordinal scale. To profile the patients based on their depression and anxiety states, the cluster analysis involved two variables, namely, the total SDS and PASS-20 scores. Second, a two-step cluster analysis was conducted. Ward’s method was performed to determine the number of clusters [21]. As an agglomerative method, it clusters similar elements, minimizing the variance within clusters at each stage of grouping. Ward’s method uses a data-driven approach instead of an arbitrary choice to develop the optimum number of clusters [22]. K-means analysis was conducted after confirming the number of clusters by pre-clustering. K-means analysis is less sensitive to outliers than other clustering methods, maximizes inter-group differences, minimizes intra-group differences, and separates observations into uniform groups [23]. The two-step clustering method is the most reliable approach for detecting the number of sub-groups, classifying observations into groups, and ensuring replicability [24]. Concurrent with the aim of our study, previous studies have shown that the two-step clustering method combining hierarchical and non-hierarchical procedures is most effective in identifying patterns of illness representations across individuals [25]. Canonical discriminant analysis was performed to determine whether inter-clusters were adequately differentiated in the selected cluster solution. Finally, differences between clusters for each variable, including the four subscales of PASS-20, were confirmed using the Kruskal-Wallis one-way analysis of variance (ANOVA) followed by the multiple-comparison test (post hoc Mann-Whitney U test) and chi-squared test or Fisher’s exact test with Bonferroni correction. Statistical analyses, cluster analyses, canonical discriminant analysis, and multiple comparisons were conducted using Statistical Package for Social Science version 27.0 J software (IBM SPSS Statistics for Windows, IBM Corp., Armonk, NY). The Kruskal-Wallis ANOVA test was performed using R version 4.1.2 (R Foundation for Statistical Computing, Vienna, Austria). The significance level was set at p = 0.05 in cluster analyses and the Kruskal-Wallis ANOVA test and at p = 0.017 in multiple comparisons. The results of descriptive statistics are expressed as median (interquartile range (IQR)) unless otherwise noted.

## Results

Dataset characteristics

Preoperative descriptive results for the sociodemographic, disease-related, physical, psychological, and disability outcomes are listed in Table 1. The median (IQR) age was 65.0 (62.0-76.0) years, and the male-to-female ratio was 1:2. Among disease-related outcomes, the median (IQR) symptom duration was 32.0 (11.0-62.0) months. Median (IQR) Padua classification of the electrophysiological severity of the median nerve was 4.0 (4.0-5.0), indicating moderate severity. In the assessment of physiological outcomes, the median (IQR) grip strength, key pinch strength, and pulp-pinch strength (kgf) were 16.0 (12.0-26.0), 5.8 (4.0-7.5), and 4.5 (2.5-6.0), respectively. The median (IQR) results for tactile threshold (g/mm^2^) and static two-point discrimination sensation (mm) were 5.0 (4.0-5.0) and 5.0 (5.0-8.0), respectively. The median (IQR) VAS score for pain and NRS score for numbness were 20.0 (5.0-40.0) and 5.0 (3.0-7.0), respectively.

**Table 1:** Descriptive results in this population (n = 65) *The results were described as the number of patients. B, bilateral; L, left; NRS, numerical rating scale; PASS-20, the Japanese version of the Pain Anxiety Symptom Scale-20; R, right; SDS, Self-Rating Depression Scale (Japanese version); VAS, visual analog scale

	Median	Quartile	
		First	Third
Sociodemographic outcome			
Age (years)	65.0	62.0	76.0
Sex (M/F) (n)	22/43^*^	-	-
Disease-related outcome			
Symptom duration (months)	32.0	11.0	62.0
Operative side (R/L) (n)	36/29^*^	-	-
Affected side (R/L/B) (n)	9/6/50^*^	-	-
Padua classification (grade)	4	4	5
Physical outcome			
Grip strength (kgf)	16.0	12.0	26.0
Key pinch strength (kgf)	5.8	4.0	7.5
Pulp pinch strength (kgf)	4.5	2.5	6.0
Tactile threshold (g/mm^2^)	5.0	4.0	5.0
Two-point discrimination sensation (mm)	5.0	5.0	8.0
VAS for pain (mm)	20.0	5.0	40.0
NRS for numbness (score)	5.0	3.0	7.0
Psychological outcome			
SDS (score)	36.0	33.0	40.0
PASS-20 (score)	14.0	9.0	41.0
Cognitive anxiety (score)	5.0	1.0	10.0
Escape/avoidance (score)	7.0	3.0	13.0
Fear (score)	2.0	0.0	6.0
Physiological anxiety (score)	1.0	0.0	3.0
Behavioral outcome			
Hand10 (score)	30.0	10.0	44.0

In the assessment of psychological states, which was considered to be an important outcome in this study, the median (IQR) SDS and PASS-20 scores were 36.0 (32.0-41.0) and 14.0 (9.0-40.0) points, respectively. Among median (IQR) PASS-20 subscales scores, the scores for cognitive anxiety, escape/avoidance, fear, and physiological anxiety were 5.0 (1.0-10.0), 7.0 (3.0-13.0), 2.0 (0.0-6.0), and 1.0 (0.0-3.0) points, respectively. The median (IQR) Hand10 score for upper extremity disability in daily life was 28.0 (10.0-47.0) points.

Cluster solution

The dendrogram using Ward’s method provided evidence for two-, three-, and five-cluster solutions with reasonable separation between clusters. The two-cluster solution was characterized as “better” and “poorer” in terms of both depression and pain anxiety. This solution showed median (IQR) SDS scores of 35.0 (32.0-38.0) and 41.0 (36.0-48.0) and median (IQR) PASS-20 total scores of 10.0 (5.0-14.0) and 47.0 (44.0-53.0). The three-cluster solution offered separation with meaningful groupings, with cluster 1 indicating the normal range of psychological functioning and showing an SDS median (IQR) score of 32.0 (26.0-35.0) score and PASS-20 median (IQR) score of 8.0 (4.0-10.0); cluster 2 indicating the marginal area of depression, albeit with median scores were within the normal range: SDS median (IQR) score, 38.0 (35.0-43.0) and PASS-20 median (IQR) score, 14.0 (13.0-15.0); and cluster 3 showing values exceeding the reference values for the corresponding measures: median (IQR) SDS score, 41.0 (36.0-48.0) and median (IQR) PASS-20 score, 47.0 (44.0-53.0). The five-cluster solution further divided cluster 1 of the three-cluster solution into three clusters on the basis of slight differences in anxiety scores. On the basis of an inspection of the dendrogram and psychological profiles by cluster, a three-cluster solution was selected to provide the best differentiation among clusters with meaningful groupings in terms of psychotic screening of preoperative patients with chronic pain following CTS. Canonical discriminant analysis confirmed that the three groups were adequately differentiated (p < 0.00 each).

The three-cluster solution produced by the K-means analysis was used for all subsequent analyses. The psychological profiles characterized by the clusters were a “psychologically normal cluster” (cluster 1), a “only depression cluster” (cluster 2), and a “psychologically impaired cluster” (cluster 3). The groups within the cluster solution were adequately differentiated by canonical discriminant analysis (p < 0.00 each).

Characteristics of psychological states and disability by cluster

Twenty-three patients (35.4%) were grouped in cluster 1, and 21 patients (32.3%) each were grouped in clusters 2 and 3. Chi-square tests or Kruskal-Wallis one-way ANOVAs showed no statistically significant differences in all comparisons among sociodemographic, disease-related, and physical outcomes (Table 2).

**Table 2:** Comparisons of sociodemographic, disease-related, and physical data between cluster 1 (psychologically normal), cluster 2 (only depression), and cluster 3 (psychologically impaired) χ^2^ tests or Kruskal-Wallis one-way analysis of variance (ANOVA), p < 0.017. ^†^The results were described as the number of patients. *The results of the χ^2^ test were shown as the t values with asterisks. The results of ANOVA were shown as the t values without asterisks. B, bilateral; L, left; NRS, numerical rating scale; R, right; VAS, visual analog scale

	Cluster 1			Cluster 2			Cluster 3			t	p
	Median	Quartile		Median	Quartile		Median	Quartile			
		First	Third		First	Third		First	Third		
Age (years)	65	62	70	65	60	68	69	65	81	1.71	0.43
Sex (M/F) (n)	7 / 16^†^	-	-	5 / 9	-	-	5 / 9	-	-	0.16^*^	0.92
Symptom duration (months)	60	12	67	35	7	57	17	10	37	3.58	0.17
Operative side (R/L)	18/5^†^	-	-	8/6	-	-	4/10	-	-	0.16^*^	0.92
Affected side (R/L/B)	2/0/21^†^	-	-	2/0/12	-	-	1/2/11	-	-	0.16^*^	0.92
Padua classification (grade)	4	4	5	4	4	5	4	4	5	0.45	0.80
Grip strength (kgf)	20.0	13.0	25.5	15.5	10.0	22.8	23.0	6.8	32.0	1.34	0.51
Key pinch strength (kgf)	6.0	4.0	7.3	4.5	2.6	7.5	5.2	4.0	7.5	0.86	0.65
Pulp pinch strength (kgf)	5.0	3.3	6.0	3.3	2.3	6.8	4.7	2.1	6.0	0.80	0.67
Tactile threshold (g/mm^2^)	5	5	5	4	4	5	5	5	5	5.07	0.08
Two-point discrimination sensation (mm)	5	5	8	5	5	6	5	5	11	0.07	0.97
VAS for pain (mm)	15	3	33	25	11	39	24	8	68	1.75	0.42
NRS for numbness (score)	3	0	7	5	3	6	7	4	8	3.04	0.22

**Figure 1:**
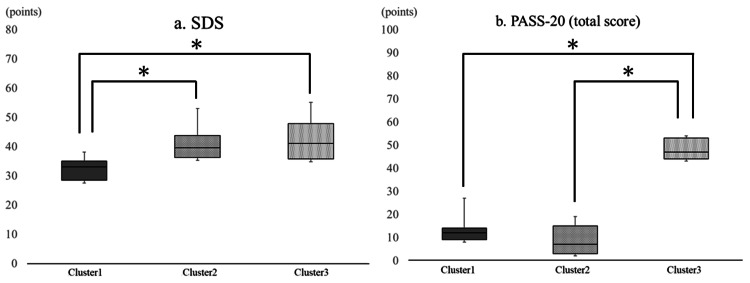
Comparisons of scores between cluster 1 (psychologically normal), cluster 2 (only depression), and cluster 3 (psychologically impaired) in (a) SDS and (b) PASS-20 *Kruskal-Wallis one-way analysis of variance (ANOVA) followed by the multiple-comparison test (post hoc Mann-Whitney U test), p < 0.017. PASS-20, Pain Anxiety Symptom Scale-20; SDS, Self-Rating Depression Scale

Psychological and disability outcomes were compared using Kruskal-Wallis one-way ANOVAs. The median (IQR) SDS scores in clusters 1, 2, and 3 were 33.0 (29.0-35.0), 40.0 (36.0-44.0), and 41.0 (36.0-48.0), respectively. SDS scores differed significantly in clusters l and 2 (p < 0.00) and clusters 1 and 3 (p < 0.00) but not in clusters 2 and 3 (p = 0.34). The median (IQR) PASS-20 total scores in clusters 1, 2, and 3 were 12.0 (9.0-14.0), 7.0 (3.0-15.0), and 47.0 (44.0-53.0), respectively. Clusters 1 and 2 did not show significant differences in the PASS-20 total score (p = 0.26), but clusters 1 and 3 and clusters 2 and 3 showed significant differences in the scores (p < 0.00) (Figure 1).

**Figure 2:**
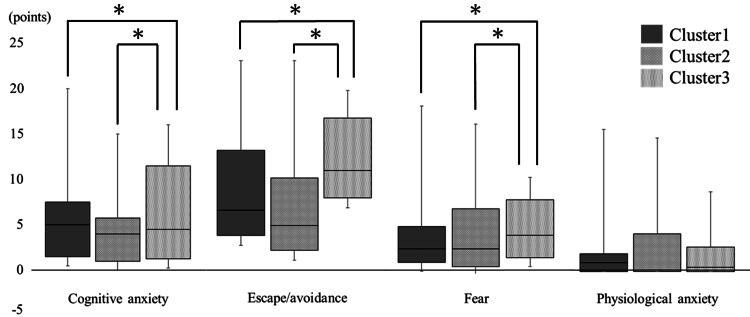
Comparisons of scores between cluster 1 (psychologically normal), cluster 2 (only depression), and cluster 3 (psychologically impaired) in each PASS-20 subscale *Kruskal-Wallis one-way analysis of variance (ANOVA) followed by the multiple-comparison test (post hoc Mann-Whitney U test), p < 0.017.

**Figure 3:**
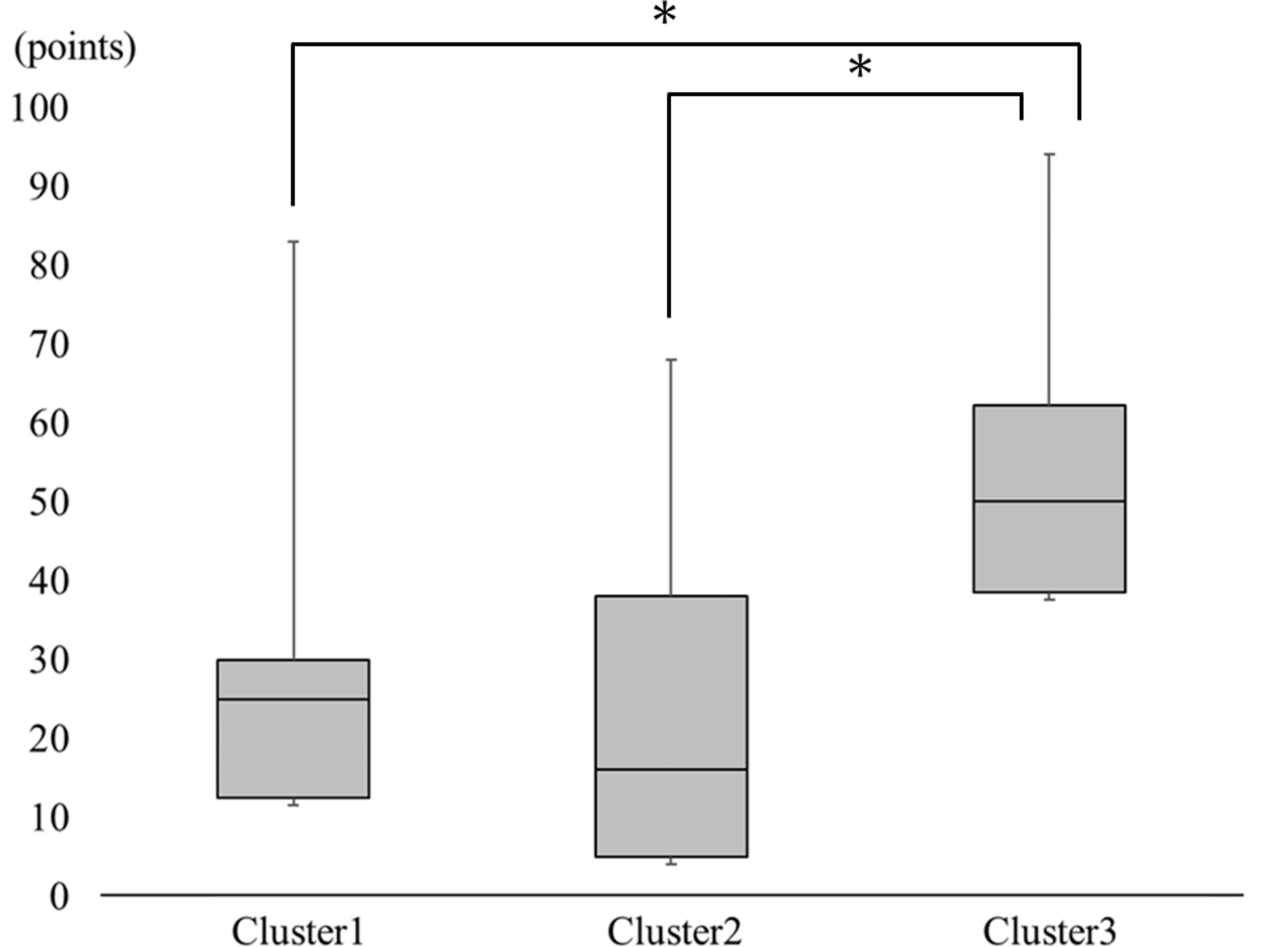
Comparisons of scores between cluster 1 (psychologically normal), cluster 2 (only depression), and cluster 3 (psychologically impaired) in Hand10 *Kruskal-Wallis one-way analysis of variance (ANOVA) followed by the multiple-comparison test (post hoc Mann-Whitney U test), p < 0.017.

The results of multiple comparisons of the four subscales of the PASS-20 and Hand10 scales are shown below. The median (IQR) PASS-20 cognitive anxiety score in clusters 1, 2, and 3 was 5.0 (2.0-8.0), 4.0 (1.0-6.0), and 5.0 (1.0-12.0), respectively. The median (IQR) escape/avoidance scores in clusters 1, 2, and 3 were 6.0 (4.0-12.0), 5.0 (2.0-9.0), and 10.0 (7.0-15.0), respectively. The median (IQR) fear scores in clusters 1, 2, and 3 were 2.0 (1.0-5.0), 2.0 (0.0-7.0), and 4.0 (1.0-8.0), respectively. Median (IQR) physiological anxiety scores in clusters 1, 2, and 3 were 1.0 (0.0-2.0), 0.0 (0.0-4.0), and 1.0 (0.0-3.0), respectively. For all subscales of the PASS-20, clusters 1 and 2 did not show statistical differences (p = 0.03-0.55). Subscales between clusters 1 and 3 and clusters 2 and 3 had statistically significant differences (p < 0.00 in each), except for physiological anxiety (p = 0.61 and 0.44, respectively) (Figure 2). The median (IQR) Hand10 scores in clusters 1, 2, and 3 were 25.0 (13.0-30.0), 16.0 (5.0-38.0), and 50.0 (39.0-62.0), respectively. In the comparisons of Hand10 scores, clusters 1 and 2 did not show statistical differences (p = 0.38), but clusters 1 and 3 and clusters 2 and 3 showed statistically significant differences (p < 0.00 each) (Figure 3).

## Discussion

This study examined depression, anxiety, demographics, physical parameters, and upper extremity disability among the patients awaiting surgery for CTS. The patients were classified into three groups. Our hypothesis that the cluster was divided into three groups was consistent with these results. Although clusters 1 and 2 were divided by the presence of depression, there was no statistically significant difference between the sociodemographic, physical, and disability outcomes in either cluster. Cluster 3 included both depression and anxiety, and the Hand10 score in cluster 3 was significantly higher than those in clusters 1 and 2. This study provides novel information on the identification of subgroups in patients with CTS before operation and its relevant upper extremity disability. These findings support the evidence of clinical impact of depression and anxiety in patients with CTS.

The sociodemographic outcomes were not significantly different among the three clusters. A recent review of CTS describes that the most common age for CTS is 50-54 years, followed by 75-84 years [26]. CTS occurs more frequently in women, although the exact male-to-female ratio varies among studies. Therefore, the target population is representative of the CTS group.

The physical and sociodemographic outcomes did not differ significantly among the three clusters. This suggests that muscle strength, tactile sensation, two-point discrimination sense, and intensities of pain and numbness were not affected by depression, anxiety, or their concurrent occurrence. This might seem unexpected because depression or anxiety results in greater improvement, at least in terms of pain [27]. Further studies are needed to determine the causal relationship between psychological and physical functions, as multiple factors, such as consciousness [28] and attentional control [29], are involved during the examination and plastic changes in the central nervous system.

Cluster 3 showed a more severe disability in the upper extremities than the other clusters. Regarding the difference in Hand10 scores, the difference between clusters 3 and 1 was 25 points, and the difference between clusters 3 and 2 was 34 points. This difference suggests that patients with CTS can engage in physical inactivity in their daily lives. This is because there are randomized controlled trials (RCTs) that exercise-based therapy, including home exercises, significantly improves the score of the patient-reported outcome measures [30]. Additionally, there is a possibility that the cognitive disturbance regarding symptoms may be associated with inactivity. Therefore, our results strengthen the evidence supporting the importance of preoperative assessments and conservative therapies from psycho-cognitive perspectives.

Study limitations

This study had some limitations. First, this was a retrospective study. However, given that the data were acquired from those in a prospective study, the risk of unrecognized bias in this study may be lower than the bias risk caused by the use of data from regular medical care alone. Second, we only assessed the psychological states at a single time point, that is, a more limited scene two weeks before the operation. Such static assessments of psychological states do not guarantee that the results for the single time point in this study are equivalent to the results assessed by other time points, such as another scene at two weeks or any other days before the operation. Therefore, future studies should examine the stability of the psychological clusters over time. If clusters represent stable symptom profiles, these groupings can be used to study associations with neurobiological measures. Furthermore, their predictive validity can be studied in terms of their clinical and functional outcomes. The identification of stable, homogeneous grouping of patients by symptom dimensions may lead to a better understanding of the pathophysiology of these illnesses and to better diagnostic procedures and treatments to target these key symptoms [31]. Third, we did not survey cognitive disturbances such as catastrophic thinking. The systematic review of catastrophic thinking is associated with higher dysfunction in 100% of the studies that considered prognostic factors [6]. Regarding upper extremity disability due to inactivity, we also had controversial findings that no difference between groups in disease duration and muscle strengths means the need to perform further study in the future. Finally, Dalmaijer et al. [32] describe that a sample size should be aimed from the n = 20 to the n = 30 per expected subgroup in cluster analysis. Our sample size was achieved to the recommendation because our cluster consisted of a minimum of 21 patients.

## Conclusions

Our findings provide evidence for the detection of depression and anxiety in patients with CTS before operation. More than one-third of the participants were grouped as showing simultaneous psychological impairment (existence of both depression and anxiety) in this study. We consider that exploring patients’ emotional experiences during the preoperative period is an important research direction in the era of modern elective surgery.
